# Malignant Growths

**Published:** 1894-04-14

**Authors:** 


### Progress in Surgery.

### MALIGNANT GROWTHS.

*Sarcoma of the Breast.*—An account of sarcoma of the breast based on thirty cases studied by W. Roger Williams shows that the liability of the female breast to this form of growth is below the average for the body generally. The author was struck by its great rarity compared with cancer. Of 2,397 consecutive neoplasms of the female breast 77·7 per cent. were cancers, while only 3·9 per cent. were sarcomas, and he states that this shows a much greater stability of the paroplastic as compared with the archiplastic elements of the gland. The great majority of mammary sarcoma arise in the immediate vicinity of the small ducts (adeno-sarcoma; only rarely do they originate elsewhere (pure sarcoma). An analysis of twelve cases of cystic adeno-sarcoma shows that the mean average was 44·8. Of nine primary cases six were married and three single. Of seven who had lived in wedlock, two were barren and never pregnant. Of eight cases there was a history of injury in two only. An analysis of seven non-cystic cases gave a mean age of 36·4 years, there was history of injury in none.

*Adeno-Sarcoma.*—The great majority consisted mainly of round or spindle-celled structures. What gives a certain peculiarity to sarcomas of the breast is the frequency with which glandular elements are incorporated in their structure. As in mammary cancer, the initial lesion in these growths generally is a small, hard, solitary nodule. Of the seventeen primary cases in his list, in only one did the first obvious manifestation consist of two distinct nodules. Of 156 cases Gross found multiple nodules in seven. In women cancer and non-malignant neoplasms arise more frequently from the left than from the right breast. Williams' statistics tend to show that the opposite holds good in regard to sarcoma. The non-cystic growths usually present as lobulated encapsuled solid tumours, of ovoid or glandular shape, varying in size from a walnut to a hen's egg. They are generally of firm and elastic consistence rather than hard. In the cystic form of the disease large, irregular, globular tumours are met with, which sometimes attain immense size. They are markedly lobulated, and their various subdivisions are irregularly nodulated. Occasionally they acquire a pedunculated form.

*Pure Sarcoma.*—Pure sarcomata seldom originate such large tumours as the adenoid variety, moreover, the former are generally less lobulated and bossy than the latter. Williams gives valuable facts in reference alveolar sarcoma, melanoma, myxoma, veloid, and sarcoma of the male breast, but for these and the other points discussed in the monograph we must refer the reader to the original communications.

*Sarcoma of the Nasal Septum.*—Dr. A. d'Aguanno,[2] of Palermo, reports a case of sarcoma of the nasal septum in a girl eleven years of age. A month before he saw the case a minute tumour was detected upon the right side of the septum, near the orifice. Within the month it had grown to the size of a large haricot bean. It was implanted transversely on its posterior half only; its surface was rugous, and it bled upon being touched with a probe. The free portion was excised with the cold snare, and the electric cautery was employed to destroy the sessile portion. Some subsequent cauterisations were necessary. At the end of two months there had been no recurrence. On microscopic examination by the professor of pathological anatomy at Palermo, Dr. Sirena, the growth was found to be a polymorphous sarcoma, lympho and fuso-cellular.

A case of *Primary Sarcoma of a Child's Liver* is reported by E. R. Axtell,[3] a rare situation for primary sarcomatous growths.

[1] Med. Chron, Feb. and Mar., 1894. [2] Ann. des Mal. de l'Oreille, 1893, xix., No. 9; Amer. Journ. Med. Sc., Jan., 1894. [3] New York Med. Rec., Mar. 3, 1894.

